# Supplementary material for: Type X strains of Toxoplasma gondii are virulent for southern sea otters (Enhydra lutris nereis) and present in felids from nearby watersheds
Source: Proc Biol Sci. 2019 Aug 21;286(1909):20191334. doi: 10.1098/rspb.2019.1334 (PMC6732395; doi:10.1098/rspb.2019.1334)
Supplement: Supplementary tables [file rspb20191334supp1.docx]

**Supplementary material**

**Table S1**. Selected loci used for genotyping *Toxoplasma gondii* isolates obtained from brains of infected sea otters (n=135) in California between 1998-2015. Genotyping was performed using virtual restriction fragment length polymorphism (RFLP) and Multi-locus sequence typing (MLST). Initially 13 loci were evaluated for the initial screening (N=29 isolates), and the remainder of the isolates were then processed at six selected loci based on their ability to discriminate between genotypes II and X and presence of additional single nucleotide polymorphisms (SNPs) that allowed higher resolution genotype differentiation.

|  |  |  | **RFLP** | |  | **MLST** | | |  |  |
| --- | --- | --- | --- | --- | --- | --- | --- | --- | --- | --- |
| **Sample set** | **Locus** |  | **Genotype differentiation** ^a^ | **Restriction sites** |  | **Genotype differentiation** | **SNP** ^b^  **no.** | **Sequence quality** |  | **References** |
| All isolates  N=135 | B1 |  | I; II/III; X | 2 |  | I; II/III; X | 2 | Good |  | [1] |
|  | L358 |  | I/X; II; III | 6 |  | I/X; II; III | 1 | Good |  | [2, 3] |
|  | PK1 |  | I; II/X; III | 6 |  | I; II; III; X | 4 | Good |  | [2, 3] |
|  | SAG1 |  | I; II/III; X | 2 |  | I; II/III; X | 0 | Good |  | [2, 3] |
|  | BTUB |  | I; II/X; III | 3 |  | I; II; III; X | 1 | Good |  | [2, 3] |
|  | GRA6 |  | I; II/X; III | 2 |  | I; II; III; X | 2 | Good |  | [2, 3] |
| Initial screening  N=29 | SAG3 |  | I; II/X; III | 3 |  | Same as RFLP | 0 | Good |  | [4] |
|  | 3'SAG2 |  | I/III; II/X | 1 |  |  | 0 | Good |  | [5] |
|  | 5'SAG2 |  | I/II/X; III | 1 |  |  | 0 | Good |  | [5][3] |
|  | AltSAG2 |  | I/III; II/X | 3 |  |  | 0 | Good |  | [2, 3] |
|  | C22-8 |  | I; II/X; III | 4 |  |  | 0 | Good |  | [2, 3] |
|  | C29-2 |  | I; II/X; III | 3 |  |  | NA^c^ | Poor |  | [2, 3] |
|  | Apico |  | I/X; II; III | 5 |  |  | 0 | Fair |  | [3] |

^a^ Genotypes that can be differentiated are delineated with a semicolon (;), while those that cannot are indicated with a slash (/)

^b^ Single nucleotide polymorphism sites in addition to those that yield cleaving sites via restriction enzymes in the RFLP approach

^c^ not assessed - sequence polymorphisms for C29-2 could not be determined due to poor sequence quality

**References**

1. Grigg M.E., Boothroyd J.C. 2001 Rapid identification of virulent type I strains of the protozoan pathogen *Toxoplasma gondii* by PCR-restriction fragment length polymorphism analysis at the B1 gene. *J Clin Microbiol* **39**(1), 398-400. (doi:10.1128/jcm.39.1.398-400.2001).

2. Khan A., Taylor S., Su C., Mackey A.J., Boyle J., Cole R., Glover D., Tang K., Paulsen I.T., Berriman M., et al. 2005 Composite genome map and recombination parameters derived from three archetypal lineages of *Toxoplasma gondii*. *Nucleic Acids Res* **33**(9), 2980-2992. (doi:10.1093/nar/gki604).

3. Su C., Zhang X., Dubey J.P. 2006 Genotyping of *Toxoplasma gondii* by multilocus PCR-RFLP markers: a high resolution and simple method for identification of parasites. *Int J Parasitol* **36**(7), 841-848. (doi:10.1016/j.ijpara.2006.03.003).

4. Grigg M.E., Ganatra J., Boothroyd J.C., Margolis T.P. 2001 Unusual abundance of atypical strains associated with human ocular toxoplasmosis. *J Infect Dis* **184**(5), 633-639. (doi:10.1086/322800).

5. Howe D.K., Honore S., Derouin F., Sibley L.D. 1997 Determination of genotypes of *Toxoplasma gondii* strains isolated from patients with toxoplasmosis. *J Clin Microbiol* **35**(6), 1411-1414.

**Table S2.** Risk factors for diagnosis of toxoplasmosis as a primary cause of death for *Toxoplasma gondii*-infected, minimally decomposed, necropsied southern sea otters from central California, 1998-2008.

| Risk Factor  (reference category) | Adjusted  Odds Ratio | 95% Confidence Interval | *p*-value |
| --- | --- | --- | --- |
| **RFLP genotype**  (Type II or Type II/X mixed)  Type X (ToxoDB #5)  **Season**  (Dry)  Wet ^a^  **Year**  (1998) | 1.00  29.22  1.00  10.28  1.00 | -  (1.38 – 620.39)  -  (1.45 – 73.02)  - | -  0.03  -  0.02  - |
| 1999 | 0.05 | (0.00 – 2.75) | 0.14 |
| 2000  2001 | 0.50  0.08 | (0.01 – 34.67)  (0.00 – 2.32) | 0.75  0.14 |
| 2002  2003  2004  2005  2006  2007  2008 | 0.05  0.01  0.74  0.04  0.02  0.01  0.65 | (0.00 – 3.04)  (0.00 – 0.71)  (0.06– 10.00)  (0.00 – 1.02)  (0.00 – 0.74)  (0.00 - 0.73)  (0.04 – 11.61) | 0.15  0.03  0.82  0.05  0.03  0.03  0.76 |

^a^ Season of sampling for each sea otter was classified as ‘wet’ or ‘dry’, with animals collected from December to May considered “wet season” samples, when rain-driven runoff in California is at its highest. Seasonal delineation was based on increased river flows of central California coastal streams due to rainfall patterns typically occurring during winter months in the Western United States (https://waterdata.usgs.gov).

**Table S3**. Univariable analysis for risk factors associated with sea otter (n=116) deaths due to *Toxoplasma gondii* infection as a primary cause. (Reference (0) = *T. gondii* not implicated or contributing to death). Predictor variables with significance of *p* <0.2 (bolded) were further considered in multivariable regression models (Table 4). Analysis was performed using bias reduced general linear regression models in R.

| **Risk factor (reference)** | **Odds**  **ratio** | **95% CI** | ***p*-value** |
| --- | --- | --- | --- |
| Genotype (RFLP Types II or mixed II/X)  Type X | 8.4 | 0.5 – 155.5 | **0.15** |
| Sample season (dry)  Wet ^a^ | 3.2 | 0.8 – 13.5 | **0.12** |
| Sample year (1998)  1999 | 0.2 | 0.0 – 7.0 | 0.38 |
| 2000 | 0.9 | 0.0 – 56.4 | 0.95 |
| 2001 | 0.8 | 0.1 – 10.7 | 0.84 |
| 2002 | 0.3 | 0.0 – 9.1 | 0.45 |
| 2003 | 0.1 | 0.0 – 3.7 | 0.22 |
| 2004 | 4.3 | 0.5 – 37.9 | **0.19** |
| 2005 | 0.5 | 0.0 – 5.9 | 0.54 |
| 2006 | 0.1 | 0.0 – 3.0 | **0.18** |
| 2007 | 0.2 | 0.0 – 7.9 | 0.41 |
| 2008 | 2.0 | 0.2 – 23.6 | 0.59 |
| Age - Binary (Subadults)  Adults and aged adults | 0.2 | 0.1 – 0.7 | **0.01** |
| Age - Categorical (Subadults)  Adults  Aged adults | 0.3  0.1 | 0.1 – 0.9  0 – 1.5 | **0.03**  **0.09** |
| Subcutaneous fat (None or scant)  Fair, moderate or abundant | 0.5 | 0.1 – 1.7 | 0.24 |
| Lymphadenopathy - Binary (Absent)  Present | 7.1 | 1.9 – 26.4 | **<0.01** |
| Lymphadenopathy - Categorical (Absent)  Mild  Moderate or marked | 5.5  9.5 | 1.2 – 25.2  2.1 – 42.2 | **0.03**  **<0.01** |
| Splenic lymphoid nodular hyperplasia – Binary (Absent)  Present | 2.2 | 0.6 – 9.0 | 0.26 |
|  |  |  |  |

^a^ Season of sampling for each sea otter was classified as ‘wet’ or ‘dry’, with animals collected from December to May considered “wet season” samples, when rain-driven runoff in California is at its highest. Seasonal delineation was based on increased river flows of central California coastal streams due to rainfall patterns typically occurring during winter months in the Western United States (https://waterdata.usgs.gov).

**Table S4.** Univariable analysis for risk factors associated with presence of Type X RFLP *Toxoplasma gondii* genotype in sea otters (n=135). (Reference (0) = Type II or mixed II/X genotype). Predictor variables with significance of *p* <0.2 (bolded) were further considered in multivariable regression models, but no significant associations were found. Analysis was performed using bias-reduced general linear regression models in R.

| **Predictor risk factor (reference)** | **Odds**  **ratio** | **95% CI** | ***p*-value** |
| --- | --- | --- | --- |
| *Toxoplasma* as a cause of death (not associated or contributing to death)  Primary cause of death | 8.4 | 0.4 – 165.4 | **0.16** |
| Sample season (dry)  Wet | 1.0 | 0.5 – 2.2 | 0.97 |
| Sample year (1998)  1999 | 1.4 | 0.2 – 11.0 | 0.72 |
| 2000 | 1.8 | 0.1 – 24.6 | 0.65 |
| 2001 | 1.3 | 0.2 – 8.9 | 0.79 |
| 2002 | 3.0 | 0.4 – 23.8 | 0.31 |
| 2003 | 2.2 | 0.4 – 13.2 | 0.39 |
| 2004 | 3.2 | 0.5 – 21.9 | 0.23 |
| 2005 | 2.3 | 0.4 – 14.9 | 0.37 |
| 2006 | 2.1 | 0.4 – 12.1 | 0.41 |
| 2007 | 14.8 | 0.5 – 420.8 | **0.12** |
| 2008 | 2.9 | 0.2 – 33.3 | 0.40 |
| Age - Binary (Pups and subadults)  Adults and aged adults | 0.7 | 0.3 – 1.9 | 0.51 |
| Age - Categorical (Pups and subadults)  Adults  Aged adults | 0.8  0.4 | 0.3 – 2.3  0.1 – 1.4 | 0.73  **0.14** |
| Subcutaneous fat (none or scant)  Fair, moderate or abundant | 1.1 | 0.4 – 2.5 | 0.91 |
| Neuropil inflammation (none, mild, or moderate)^b^  Marked | 2.0 | 0.3 – 12.7 | 0.47 |
| Brain perivascular inflammation (none, mild, or moderate)  Marked | 1.2 | 0.4 – 3.8 | 0.77 |
| Meningeal inflammation (none, mild, or moderate)  Marked | 1.2 | 0.4 – 3.9 | 0.73 |
| Myocardial inflammation (none, mild, or moderate)  Marked | 0.6 | 0.2 – 2.5 | 0.50 |
| Lymphadenopathy - Binary (Absent)  Present | 1.9 | 0.7 – 5.6 | 0.22 |
| Lymphadenopathy - Categorical (Absent)  Mild  Moderate or marked | 1.6  2.0 | 0.4 – 5.8  0.5 – 8.6 | 0.48  0.37 |
| Splenic lymphoid nodular hyperplasia – Binary (Absent)  Present | 1.3 | 0.4 – 4.9 | 0.67 |
| Parasites CNS (Not visualized)^c^  Visualized | 1.3 | 0.5 – 3.4 | 0.55 |
|  |  |  |  |
| Parasites Myocardium (not visualized)  Visualized | 2.5 | 0.6 – 10.4 | 0.21 |
|  |  |  |  |

^a^ Season of sampling for each sea otter was classified as ‘wet’ or ‘dry’, with animals collected from December to May considered “wet season” samples, when rain-driven runoff in California is at its highest. Seasonal delineation was based on increased river flows of central California coastal streams due to rainfall patterns typically occurring during winter months in the Western United States (https://waterdata.usgs.gov).

^b^ Inflammation of each tissue was categorized as marked, moderate, mild, or none on pathology assessment. Although results for marked inflammation vs. other are presented here, univariable tests were also performed for additional categorical comparisons, with none found to have significant associations with RFLP genotype (e.g. none vs. other; none and mild vs. marked and moderate; none and mild vs. moderate or marked).

^c^ Parasites in muscle were also evaluated during histopathology assessments. However, parasites were only detected in the muscle of five animals, which did not allow robust univariable odds ratio estimates.

**Table S5**. Multi-locus sequence typing (MLST) of *Toxoplasma gondii* isolates from reference strains, terrestrial felids and southern sea otters demonstrated a Type X variant strain at the B1 gene, with a single nucleotide polymorphism at nucleotide position 189. A double peak in the chromatogram indicated mixed C/G alleles at this multi-copy gene. The same X variant strain was found in two feral cats and one bobcat from the same coastal region of California [17] (figure 4b). This X variant strain was isolated from brain tissue of five sea otters with toxoplasmosis as a primary cause of death.

|  |  | **Nucleotide position** | | | |  |
| --- | --- | --- | --- | --- | --- | --- |
|  |  | **360** | **366*** | **504*** |  | |
| **Sample Type**  **and ID** | **Consensus** | **C** | **T/C** | **C/G** | **RFLP Allele Type** | |
| Reference strains |  | . | T | G |  | |
| Type I (RH) |  | . | T | G | I | |
| Type II (ME49) |  | . | . | . | II/III | |
| Type III (CTG) |  | . | . | . | II/III | |
| Type X^a^ (Bobcat 4) |  | . | T | C | X | |
| Carnivores |  |  |  |  |  | |
| Feral cat 29^b^ |  | C/G | T | C | X | |
| Feral cat 30 |  | C/G | T | C | X | |
| Bobcat 6 |  | C/G | T | C | X | |
| Sea Otters |  | C/G | T | C | X | |
| Type X variant^c^ |  |  |  |  |  |  |
| (n=31) |  |  |  |  |  |  |

*SNP that corresponds with RFLP enzyme cleaving site. Nucleotide positions were determined by aligning all sequences to the RH Type I genotype (Genbank #AF179871).

^a^ DNA sequence from a tachyzoite-infected cell culture of brain tissue from Type X-infected Bobcat 4 identified by [17].

^b^ This B1 sequence from a Type X-infected domestic cat (FC 29) was previously described by [17]. This sequence is also consistent with Type X-infected Sea Otter 3160 [15]

^c^ Thirty-one animals (23%) had a Type X variant MLST strain at the B1 locus.
